# Antimicrobial Resistance and *in silico* Virulence Profiling of *Aliarcobacter butzleri* Strains From German Water Poultry

**DOI:** 10.3389/fmicb.2020.617685

**Published:** 2020-12-14

**Authors:** Eva Müller, Helmut Hotzel, Jörg Linde, Ingrid Hänel, Herbert Tomaso

**Affiliations:** Institute of Bacterial Infections and Zoonoses (IBIZ), Friedrich-Loeffler-Institut, Federal Research Institute for Animal Health, Jena, Germany

**Keywords:** *Aliarcobacter*, emerging pathogen, heavy metal resistance, virulence, antimicrobial resistance, whole-genome sequencing, antibiotic susceptibility

## Abstract

*Aliarcobacter butzleri* is an emerging foodborne and zoonotic pathogen that is usually transmitted via contaminated food or water. *A. butzleri* is not only the most prevalent *Aliarcobacter* species, it is also closely related to thermophilic *Campylobacter*, which have shown increasing resistance in recent years. Therefore, it is important to assess its resistance and virulence profiles. In this study, 45 *Aliarcobacter butzleri* strains from water poultry farms in Thuringia, Germany, were subjected to an antimicrobial susceptibility test using the gradient strip diffusion method and whole-genome sequencing. In the phylogenetic analysis, the genomes of the German strains showed high genetic diversity. Thirty-three isolates formed 11 subgroups containing two to six strains. The antimicrobial susceptibility testing showed that 32 strains were resistant to erythromycin, 26 to doxycycline, and 20 to tetracycline, respectively. Only two strains were resistant to ciprofloxacin, while 39 strains were resistant to streptomycin. The *in silico* prediction of the antimicrobial resistance profiles identified a large repertoire of potential resistance mechanisms. A strong correlation between a *gyr*A point mutation (Thr-85-Ile) and ciprofloxacin resistance was found in 11 strains. A partial correlation was observed between the presence of the *bla*3 gene and ampicillin resistance. *In silico* virulence profiling revealed a broad spectrum of putative virulence factors, including a complete lipid A cluster in all studied genomes.

## Introduction

The species *Aliarcobacter* (*A*.) *butzleri* (former *Arcobacter butzleri*) belongs to the genus *Aliarcobacter*, a member of the *Campylobacteraceae* family, together with *A. cryaerophilus, A. skirrowii, A. thereius, A. cibarius, A. lanthieri, A. faecis*, and *A. trophiarum* (Pérez-Cataluña et al., 2018a,b, 2019).

*A. butzleri* is an emerging foodborne and zoonotic pathogen that has been considered as a serious hazard to human health (ICMSF, 2002; Collado and Figueras, 2011; Ramees et al., 2017). In humans, *A. butzleri* infections are associated with self-limiting enteritis, diarrhea and rarely bacteremia; in animals, it has been associated with abortions, mastitis, and gastrointestinal diseases (Logan et al., 1982; Vandamme et al., 1992; Woo et al., 2001; Lau et al., 2002; Vandenberg et al., 2004; Collado and Figueras, 2011; Chieffi et al., 2020).

*A. butzleri* is the most prevalent foodborne *Aliarcobacter* species, followed in frequency by *A. cryaerophilus* and *A. skirrowii* (Lehner et al., 2005; Collado and Figueras, 2011; Ramees et al., 2017; Caruso et al., 2020). The bacteria have been found in water, vegetables, meat (especially poultry), milk, dairy products, and shellfish (Ho et al., 2006; Hausdorf et al., 2013; Yesilmen et al., 2014; Ramees et al., 2017; Fanelli et al., 2019; Caruso et al., 2020; Chieffi et al., 2020). Since *A. butzleri* is frequently isolated from food-producing animals, these animals should be considered as an important reservoir (Rahimi, 2014; Giacometti et al., 2015; Rathlavath et al., 2017; Sekhar et al., 2017; Caruso et al., 2018; Chieffi et al., 2020). However, this would require successful colonization of the intestines and contamination during the slaughter process (Chieffi et al., 2020).

The consumption of contaminated food/feed or water is the main transmission route for humans and animals. The contamination of food products is probably a consequence of unhygienic procedures (Chieffi et al., 2020). Contact from person-to-person and companion animals-to-person are also possible ways of transmission to humans (Fera et al., 2009; Collado and Figueras, 2011; Shah et al., 2012a; Giacometti et al., 2015; Ferreira et al., 2016; Ramees et al., 2017; Chieffi et al., 2020).

The antimicrobial susceptibility of *A. butzleri* has been frequently investigated phenotypically (Harrass et al., 1998; Fera et al., 2003; Houf et al., 2004; Ho et al., 2006; Vandenberg et al., 2006; Abay et al., 2012; Ferreira et al., 2013, 2017; Shah et al., 2013; Rahimi, 2014; Shirzad Aski et al., 2016; Van den Abeele et al., 2016; Pérez-Cataluña et al., 2017; Vicente-Martins et al., 2018; Fanelli et al., 2019, 2020; Parisi et al., 2019). The underlying genetic resistance mechanisms have been studied in more detail in recent years but data is still limited (Abdelbaqi et al., 2007; Merga et al., 2013a; Fanelli et al., 2019, 2020; Parisi et al., 2019; Isidro et al., 2020). In addition to virulence-associated genes that are homologs of genes previously found in *Campylobacter* (*C*.) *jejuni* (*pld*A, *mvi*N, *irg*A, *iro*E, *cia*B, *hec*A, *hec*B, *cj*1345, *cad*F, *tly*A), further potential virulence-associated genes have been discovered (Miller et al., 2007; Fanelli et al., 2019, 2020; Isidro et al., 2020). Only little is known about the resistance of *A. butzleri* to heavy metals (Fanelli et al., 2019, 2020).

Here, we report antibiotic susceptibility profiles of 45 *A. butzleri* strains isolated from water poultry in Thuringia, Germany, and present insights into their phylogeny. Furthermore, we have complemented these data with 30 *A. butzleri* genomes from public databases, and described the presence of putative antimicrobial resistance genes, potential heavy metal resistance genes and virulence-associated genes in all genomes.

## Materials and Methods

### Bacterial Strains, Culturing, and Identification

In 2016 and 2017, 188 fecal samples were collected from clinically healthy animals from seven water poultry farms in Thuringia, Germany. In detail, 88 fecal samples were collected in 2016 from five water poultry farms from 60 geese (*Anser anser*), 13 Muscovy ducks (*Cairina moschata*), and 15 mulard ducks (*Cairina moschata* × *Anas platyrhynchos domesticus*). In 2017, 100 fecal samples were gathered from 50 geese, 25 Muscovy ducks, 15 mulard ducks, and 10 Pekin ducks (*Anas platyrhynchos domesticus*) from seven water poultry farms. A veterinarian collected the fecal samples with the permission of the animal owners.

For this study, no ethical review process was required as there were no experiments with animals as defined by the German Animal Protection Law (Tierschutzgesetz) and the Animal Welfare Laboratory Animal Regulation (Tierschutz-Versuchstierverordnung).

Culturing and identification was performed as described previously (Müller et al., 2020a). Briefly, the *Aliarcobacter* isolates were cultivated in *Arcobacter* broth (Oxoid GmbH, Wesel, Germany), and then spread on plates (Mueller-Hinton-agar/5% defibrinated bovine blood; Sifin GmbH, Berlin, Germany). Nutrient broth and agar plates were supplemented with antibiotics (cefoperazone, amphotericin, and teicoplanin (CAT), SR0174, Oxoid GmbH). The incubation criteria were: 48–72 h at 30°C in microaerophilic atmosphere (5% O_2_, 10% CO_2_, and 85% N_2_). Suspicious colonies were further cultivated and subsequently identified by matrix-assisted laser desorption/ionization time-of-flight mass spectrometry (MALDI-TOF MS) as described before (El-Ashker et al., 2015; Hänel et al., 2018). DNA was purified using the High Pure PCR Template Preparation Kit (Roche Diagnostics, Mannheim, Germany) following the manufacturer's instructions and confirmation of the species identification was done with a multiplex PCR assay (Houf et al., 2000).

### Antibiotic Susceptibility Testing

Antibiotic susceptibility was determined using the gradient strip diffusion method (E-Test^TM^, bioMérieux, Nürtingen, Germany) as previously described (Müller et al., 2020a). Each strain was tested two times against erythromycin, ciprofloxacin, streptomycin, gentamicin, tetracycline, doxycycline, ampicillin, and cefotaxime. The minimum inhibitory concentration (MIC) was determined after 48 h of incubation at 30°C under microaerophilic conditions. The *A. butzleri* type strain DSM 8739 was used as a control. In this study, the cut-off values for *Campylobacter* spp. provided by EUCAST (2019) were used for erythromycin, ciprofloxacin, doxycycline, and tetracycline because currently no specific breakpoints are available for *Aliarcobacter* spp. For gentamicin, ampicillin, and cefotaxime, we used the breakpoints for *Enterobacterales* provided by EUCAST (2019). For streptomycin, the cut-off values for *Campylobacter* spp. provided by the EFSA Journal 2019 were used [European Food Safety Authority (EFSA) et al., 2019]. The bacterial strains were classified as sensitive, intermediate, or resistant.

### Urease Test

For the urease test, the following ingredients of a urea broth (Merck KgaA, Darmstadt, Germany) were added to one liter of *Arcobacter* broth: potassium hydrogen phosphate (7.63 g/L), disodium hydrogen phosphate (9.59 g/L), urea (20.0 g/L), and phenol red (0.012 g/L). The final broth was inoculated with colony material from fresh *A. butzleri* cultures and incubated at 30°C for 48 h under microaerophilic conditions to increase bacterial growth. Then, the broth was further incubated at 37°C for at least 72 h. A color change from orange to pink was considered as a positive result. The urease assay was done for the 45 *A. butzleri* strains investigated in this study as well as for two strains (16CS0817-2 and 16CS0821-2) for which culture material was available as they were previously examined by our institute (Müller et al., 2020a). Each strain was subjected to the urease assay twice.

### DNA Extraction and Whole-Genome Sequencing

DNA extraction was performed for 45 *A. butzleri* isolates as described previously (Müller et al., 2020a). Briefly, the DNA was extracted from fresh *A. butzleri* cultures using the Qiagen Genomic-tip 20/G (Qiagen GmbH, Hilden, Germany). The concentration of the double-stranded DNA (dsDNA) was examined with a Qubit 3 Fluorometer using the Qubit^TM^ dsDNA HS Assay Kit (both Invitrogen^TM^, ThermoFischer Scientific, Berlin, Germany). The Nextera XT DNA Library Preparation Kit (Illumina, Inc., San Diego, CA, United States) was used according to the manufacturer's instructions to generate a paired-end sequencing library. Whole-genome sequencing was done with an Illumina MiSeq instrument (Illumina, Inc.) generating reads of 300 base pairs (bp) in length.

### Bioinformatic Analyses

The 45 genomes sequenced in this study were analyzed together with 29 European *A. butzleri* genomes (France = 1, UK = 1, Italy = 2, Germany = 4, and Portugal = 21) (Fanelli et al., 2019; Isidro et al., 2020; Müller et al., 2020a) and the *A. butzleri* reference genome (NCTC 12481^T^, accession: GCF_900187115.1) (Table 1, Supplementary Table 1). Those additional *A. butzleri* genomes were downloaded from the NCBI GenBank database (last accessed on 08.07.2020) (Benson et al., 2013). For 24 publicly available *A. butzleri* genomes raw sequencing reads were available and downloaded from the Sequence Read Archive (SRA, last accessed on 08.07.2020) (Leinonen et al., 2011). The raw reads together with the raw data of the 45 *A. butzleri* isolates sequenced herein were *de novo* assembled. For the analyses, we used the WGSBAC pipeline (https://gitlab.com/FLI_Bioinfo/WGSBAC) as described previously (Garcia-Soto et al., 2020; Wareth et al., 2020). WGSBAC assembled the raw data using shovill version 1.0.4 (https://github.com/tseemann/shovill) after adapter trimming with Trimmomatic (Bolger et al., 2014). The quality of all assemblies was then assessed with QUAST version 5.0.2 (Gurevich et al., 2013). The software Prokka version 1.14.5 (Seemann, 2014) in default settings was used to annotate the assembled genomes.

**Table 1:** The metadata of 75 *A. butzleri* strains used in this study.

**WGS**	**Strain**	**BioProject**	**Isolation source**	**Year of isolation**	**Geographic location**	**Farm**	**Sample No**.	**References**
SRR12709624	16CS0847-3	PRJNA665330	Fecal sample (goose)	2016	Germany	A	1	This study
SRR12709623	17CS0824	PRJNA665330	Fecal sample (goose)	2017	Germany	A	2	This study
SRR12709612	17CS1193-1	PRJNA665330	Fecal sample (mulard duck)	2017	Germany	A	3	This study
SRR12709601	17CS1193-2	PRJNA665330	Fecal sample (mulard duck)	2017	Germany	A	3	This study
SRR12709635	17CS1193-3	PRJNA665330	Fecal sample (mulard duck)	2017	Germany	A	3	This study
SRR12709629	17CS1197	PRJNA665330	Fecal sample (Muscovy duck)	2017	Germany	A	4	This study
SRR12709628	17CS1197-2	PRJNA665330	Fecal sample (Muscovy duck)	2017	Germany	A	4	This study
SRR12709627	17CS1197-3	PRJNA665330	Fecal sample (Muscovy duck)	2017	Germany	A	4	This study
SRR12709626	17CS1198-1	PRJNA665330	Fecal sample (Muscovy duck)	2017	Germany	A	5	This study
SRR12709625	17CS1198-2	PRJNA665330	Fecal sample (Muscovy duck)	2017	Germany	A	5	This study
SRR12709622	17CS1198-3	PRJNA665330	Fecal sample (Muscovy duck)	2017	Germany	A	5	This study
SRR12709621	16CS0367-AR	PRJNA665330	Fecal sample (goose)	2016	Germany	B	6	This study
SRR12709620	16CS0367-1-AR	PRJNA665330	Fecal sample (goose)	2016	Germany	B	6	This study
SRR12709619	16CS0370-1	PRJNA665330	Fecal sample (goose)	2016	Germany	B	7	This study
SRR12709618	16CS0375-1	PRJNA665330	Fecal sample (goose)	2016	Germany	B	8	This study
SRR12709617	16CS0375-2	PRJNA665330	Fecal sample (goose)	2016	Germany	B	8	This study
SRR12709616	16CS0831-1-1	PRJNA665330	Fecal sample (goose)	2016	Germany	B	9	This study
SRR12709615	16CS0831-4-1	PRJNA665330	Fecal sample (goose)	2016	Germany	B	9	This study
SRR12709614	17CS1200-1	PRJNA665330	Fecal sample (Pekin duck)	2017	Germany	B	10	This study
SRR12709613	17CS1201	PRJNA665330	Fecal sample (Pekin duck)	2017	Germany	B	11	This study
SRR12709611	17CS1205-1	PRJNA665330	Fecal sample (Pekin duck)	2017	Germany	B	12	This study
SRR12709610	17CS1205-2	PRJNA665330	Fecal sample (Pekin duck)	2017	Germany	B	12	This study
SRR12709609	17CS1206-1	PRJNA665330	Fecal sample (Pekin duck)	2017	Germany	B	13	This study
SRR12709608	17CS1208-3	PRJNA665330	Fecal sample (Pekin duck)	2017	Germany	B	14	This study
SRR12709607	17CS1167	PRJNA665330	Fecal sample (goose)	2017	Germany	C	15	This study
SRR12709606	17CS1168	PRJNA665330	Fecal sample (goose)	2017	Germany	C	16	This study
SRR12709605	17CS1056	PRJNA665330	Fecal sample (goose)	2017	Germany	D	17	This study
SRR12709604	17CS1066	PRJNA665330	Fecal sample (Muscovy duck)	2017	Germany	D	18	This study
SRR12709603	17CS1067	PRJNA665330	Fecal sample (Muscovy duck)	2017	Germany	D	19	This study
SRR12709602	17CS1068-1	PRJNA665330	Fecal sample (Muscovy duck)	2017	Germany	D	20	This study
SRR12709600	17CS1068-2	PRJNA665330	Fecal sample (Muscovy duck)	2017	Germany	D	20	This study
SRR12709599	17CS0916-1	PRJNA665330	Fecal sample (Muscovy duck)	2017	Germany	E	21	This study
SRR12709598	17CS0916-2	PRJNA665330	Fecal sample (Muscovy duck)	2017	Germany	E	21	This study
SRR12709597	16CS0857-1	PRJNA665330	Fecal sample (goose)	2016	Germany	F	22	This study
SRR12709596	16CS0861-1	PRJNA665330	Fecal sample (goose)	2016	Germany	F	23	This study
SRR12709595	17CS1092-1	PRJNA665330	Fecal sample (goose)	2017	Germany	F	24	This study
SRR12709594	17CS1092-2	PRJNA665330	Fecal sample (goose)	2017	Germany	F	24	This study
SRR12709593	17CS1095	PRJNA665330	Fecal sample (goose)	2017	Germany	F	25	This study
SRR12709637	16CS0815-2	PRJNA665330	Fecal sample (goose)	2016	Germany	G	26	This study
SRR12709636	16CS0820-1	PRJNA665330	Fecal sample (Muscovy duck)	2016	Germany	G	27	[1], this study
SRR12709634	16CS0820-2	PRJNA665330	Fecal sample (Muscovy duck)	2016	Germany	G	27	This study
SRR12709633	16CS0823-1	PRJNA665330	Fecal sample (Muscovy duck)	2016	Germany	G	28	This study
SRR12709632	16CS0823-2	PRJNA665330	Fecal sample (Muscovy duck)	2016	Germany	G	28	[1], this study
SRR12709631	17CS0965-1	PRJNA665330	Fecal sample (goose)	2017	Germany	G	29	This study
SRR12709630	17CS0965-B	PRJNA665330	Fecal sample (goose)	2017	Germany	G	29	This study
SRR10215626	16CS0817-2	PRJNA575341	Fecal sample (Muscovy duck)	2016	Germany	G	–	This study
SRR10215625	16CS0821-2	PRJNA575341	Fecal sample (Muscovy duck)	2016	Germany	G	–	This study
GCF_900187115.1	NCTC 12481	PRJEB6403	Diarrheic stool (human)	1992	USA	–	–	NA, [9]^#^, [10]^§^
GCF_004283125.1	55	PRJNA489574	Mussel	2014	Italy	–	–	[2]
GCF_004283115.1	6V	PRJNA489609	Clams	2014	Italy	–	–	[2]
GCF_013363865.1	RMI	PRJNA637480	Raw milk	2018	Germany	–	–	NA
GCF_013363855.1	RMIII	PRJNA637480	Raw milk	2018	Germany	–	–	NA
GCF_000215345.2	7h1h	PRJNA67167	Fecal sample (beef)	2007	UK	–	–	[3, 6]
ERR3523212	Ab_1426_2003	PRJEB34441	Diarrheic stool (human)	2003	France	–	–	[4, 6]
ERR3523199	Ab_1711	PRJEB34441	Poultry slaughterhouse equipment surface	2011	Portugal	–	–	[5, 6]
ERR3523202	Ab_2211	PRJEB34441	Slaughterhouse surface	2011	Portugal	–	–	[5, 6]
ERR3523203	Ab_2811	PRJEB34441	Carcass neck skin (poultry)	2011	Portugal	–	–	[5, 6]
ERR3523197	Ab_3711	PRJEB34441	Caecum (poultry)	2011	Portugal	–	–	[5, 6]
ERR3523210	Ab_4211	PRJEB34441	Carcass drippings (poultry)	2011	Portugal	–	–	[5, 6]
ERR3523201	Ab_4511	PRJEB34441	Carcass drippings (flock)	2011	Portugal	–	–	[5, 6]
ERR3523204	Ab_A103	PRJEB34441	River water	2016	Portugal	–	–	[6]
ERR3523214	Ab_A111	PRJEB34441	River water	2016	Portugal	–	–	[6]
ERR3523198	Ab_CR1132	PRJEB34441	Ready-to-eat vegetables	2016	Portugal	–	–	[6, 7]
ERR3523196	Ab_CR1143	PRJEB34441	Meat (poultry)	2016	Portugal	–	–	[6, 7]
ERR3523205	Ab_CR424	PRJEB34441	Meat (poultry)	2015	Portugal	–	–	[6, 7]
ERR3523200	Ab_CR461	PRJEB34441	Meat (fish)	2015	Portugal	–	–	[6, 7]
ERR3523213	Ab_CR502	PRJEB34441	Meat (poultry)	2015	Portugal	–	–	[6, 7]
ERR3523209	Ab_CR604	PRJEB34441	Meat (beef)	2015	Portugal	–	–	[6, 7]
ERR3523206	Ab_CR641	PRJEB34441	Meat (poultry)	2015	Portugal	–	–	[6, 7]
ERR3523216	Ab_CR891	PRJEB34441	Meat (poultry)	2016	Portugal	–	–	[6, 7]
ERR3523215	Ab_CR892	PRJEB34441	Meat (poultry)	2016	Portugal	–	–	[6, 7]
ERR3523207	Ab_DQ20dA1	PRJEB34441	Milk (goat)	2015	Portugal	–	–	[6, 8]
ERR3523217	Ab_DQ31A1	PRJEB34441	Milk (sheep)	2015	Portugal	–	–	[6, 8]
ERR3523211	Ab_DQ40A1	PRJEB34441	Dairy plant equipment surface	2015	Portugal	–	–	[6, 8]
ERR3523208	Ab_DQ64A1	PRJEB34441	Dairy plant equipment surface	2015	Portugal	–	–	[6, 8]

*[1] - Müller et al. (2020a); [2] - Fanelli et al. (2019); [3] - Merga et al. (2013b); [4] - Ferreira et al. (2014); [5] - Ferreira et al. (2013); [6] - Isidro et al. (2020); [7] - Vicente-Martins et al. (2018); [8] - Ferreira et al. (2017); [9] - Miller et al. (2007); [10] - Miller et al. (2009); NA, not available*;

# *antimicrobial susceptibility values and result of the urease assay were taken from reference [9]*;

§ *results of the MLST analysis were taken from reference [10]*.

To confirm the species identity, taxonomic classification was performed using Kraken2 version 2.0.7_beta with the database Kraken2DB (Wood et al., 2019). The average nucleotide identity (ANI) was calculated using pyani version 0.2.9 (module ANIblastall) (Pritchard et al., 2016) for all *A. butzleri* strains (including the reference genome NCTC 12481^T^) in comparison to the reference genomes of *A. cryaerophilus* ATCC 43158^T^ (accession: GCF_003660105.1) and *A. trophiarum* LMG 25534^T^ (accession: GCF_003355515.1) as well as to the out-group genome *C. jejuni* subsp. *jejuni* NCTC 11168 (accession: GCF_000009085.1), which were downloaded from the NCBI repository. *In silico* DNA-DNA hybridization (DDH) was done using the Genome-to-Genome Distance Calculator (GGDC) software (available at https://ggdc.dsmz.de). In this study, the recommended formula 2 was used for analysis (Meier-Kolthoff et al., 2013). Multilocus sequence typing (MLST) based on the whole-genome sequences was performed using the mlst software version 2.19.0 (https://github.com/tseemann/mlst) and the PubMLST database (pubmlst.org/arcobacter; last accessed on 27.05.2020) (Jolley and Maiden, 2010). Phylogenetic analyses were performed using Roary version 3.13.0 (Page et al., 2015) as previously applied for *A. butzleri* (Pérez-Cataluña et al., 2018b) and Snippy version 4.3.6 (https://github.com/tseemann/snippy). The phylogenetic tree, based on the Roary output, was built using FastTree version 2.1.9 (Price et al., 2009, 2010) and visualized with iTOL version 5.6.3 (Letunic and Bork, 2007).

For further investigations, two custom-made databases specific for *A. butzleri* (available at https://gitlab.com/FLI_Bioinfo_pub, Müller et al., 2020a) were used to determine the presence of potential antimicrobial and heavy metal resistance genes as well as the presence of virulence-associated genes within ABRicate version 0.8.10 (https://github.com/tseemann/abricate). A gene was considered to be present with a detection value of at least 50% coverage and 75% identity. In addition, the *gyr*A gene, the 23S rRNA gene, the *rpl*V gene and the *rpl*D gene of all *A. butzleri* genomes were extracted and analyzed using Geneious Prime® 2019.2.3 (Kearse et al., 2012) to identify any known point mutations or amino acid changes that are known to exist in *Campylobacter* spp. (Perez-Boto et al., 2010; Bolinger and Kathariou, 2017; Shen et al., 2018; Elhadidy et al., 2020).

Plasmid prediction was performed for all *A. butzleri* strains using BLASTn (Altschul et al., 1990) and the NCBI RefSeq plasmid database (https://ftp.ncbi.nlm.nih.gov/refseq/release/plasmid/). With a detection value of at least 55% coverage and 85% identity, a plasmid was considered to be present.

## Results and Discussion

### Bacterial Strains and Whole-Genome Sequencing

Out of 188 fecal samples, 29 were positive for *A. butzleri*, 10 in 2016 and 19 in 2017. These were obtained from 15 geese, eight Muscovy ducks, five Pekin ducks, and one mulard duck (Table 1). Because of the different morphology of the suspected *A. butzleri* colonies on the culture plates, one to three single colonies were picked and processed separately. In total, 45 *A. butzleri* strains were identified by MALDI-TOF MS and multiplex PCR assay. Since sufficient spectra for *A. butzleri* were available in the database, species identification by MALDI-TOF MS (scores > 2.3) was reliable (Hänel et al., 2018). The multiplex PCR assay identified the species *A. butzleri* with 100% reliability (Houf et al., 2000; Levican and Figueras, 2013).

In the present study, whole-genome sequencing of 45 *A. butzleri* strains was performed. The Illumina sequencing yielded an average number of 0.86 million reads per strain (range: 219,906–3,042,034 reads) and an average depth of coverage of 73.9-fold (range: 23 to 176-fold). For the 45 genomes, an average N50 value of 137.0 kbp (range: 22.9–255.9 kbp) and an average of 49 contigs per strain (range: 17–191 contigs) was calculated (Table 2).

**Table 2:** Sequencing statistics, assembly statistics, and annotation of 45 German *A. butzleri* strains.

**ID**	**Sequencing statistics**				**Assembly statistics**				**Annotation**		
	**Sequencing platform**	**Total number of reads (X1000)**	**Average read length (bp)**	**Coverage depth**	**Genome size**	**No. contigs**	**N50**	**GC%**	**Total CDS**	**No. rRNA**	**No. tRNA**
16CS0847-3	Illumina MiSeq	539.2	230	52	2.12 Mbp	30	114,025	26.96	2,101	3	46
17CS0824	Illumina MiSeq	917.0	208	80	2.19 Mbp	35	135,672	26.92	2,155	3	47
17CS1193-1	Illumina MiSeq	368.9	222	34	2.23 Mbp	20	201616	26.93	2,223	3	46
17CS1193-2	Illumina MiSeq	554.0	233	54	2.23 Mbp	17	237,465	26.93	2,223	3	46
17CS1193-3	Illumina MiSeq	473.0	233	46	2.19 Mbp	22	209,048	26.89	2,181	3	45
17CS1197	Illumina MiSeq	337.4	219	31	2.21 Mbp	73	63,178	27.00	2,216	3	46
17CS1197-2	Illumina MiSeq	533.7	225	50	2.21 Mbp	47	117,432	26.99	2,224	3	46
17CS1197-3	Illumina MiSeq	823.1	218	75	2.21 Mbp	47	105,309	26.99	2,225	3	46
17CS1198-1	Illumina MiSeq	885.7	245	91	2.16 Mbp	35	181,112	26.90	2,164	3	45
17CS1198-2	Illumina MiSeq	910.2	217	83	2.16 Mbp	35	181,142	26.90	2,166	3	45
17CS1198-3	Illumina MiSeq	789.5	209	69	2.16 Mbp	36	165,442	26.90	2,166	3	46
16CS0367-AR	Illumina MiSeq	1,001.3	237	99	2.19 Mbp	79	54,432	26.96	2,204	3	46
16CS0367-1-AR	Illumina MiSeq	219.9	257	23	2.20 Mbp	25	251,305	26.94	2,214	3	45
16CS0370-1	Illumina MiSeq	1,038.6	224	97	2.20 Mbp	25	251,305	26.94	2,213	3	45
16CS0375-1	Illumina MiSeq	630.7	259	68	2.20Mbp	26	251,306	26.94	2,213	3	46
16CS0375-2	Illumina MiSeq	1,009.2	215	91	2.20 Mbp	36	137,063	26.94	2,214	3	46
16CS0831-1-1	Illumina MiSeq	1,566.2	173	113	2.20 Mbp	34	157,075	26.94	2,211	3	46
16CS0831-4-1	Illumina MiSeq	759.8	231	73	2.26 Mbp	24	170,136	26.91	2,255	3	47
17CS1200-1	Illumina MiSeq	792.7	235	78	2.12 Mbp	31	186,556	26.95	2,122	3	46
17CS1201	Illumina MiSeq	1,015.7	210	89	2.12 Mbp	34	157,092	26.95	2,122	3	46
17CS1205-1	Illumina MiSeq	315.2	202	26	2.24 Mbp	61	81,235	27.06	2,311	3	47
17CS1205-2	Illumina MiSeq	823.7	222	76	2.24 Mbp	42	130,656	26.88	2,316	3	47
17CS1206-1	Illumina MiSeq	903.2	214	81	2.24 Mbp	53	103,432	26.88	2,315	3	47
17CS1208-3	Illumina MiSeq	333.4	209	29	2.24 Mbp	64	117,355	26.89	2,311	3	47
17CS1167	Illumina MiSeq	451.7	232	44	2.21 Mbp	31	117,108	26.91	2,170	3	45
17CS1168	Illumina MiSeq	1,422.1	246	147	2.17 Mbp	40	103,522	26.92	2,143	3	46
17CS1056	Illumina MiSeq	279.2	208	24	2.19 Mbp	191	22,951	26.99	2,167	3	45
17CS1066	Illumina MiSeq	459.5	265	51	2.21 Mbp	71	66,964	26.93	2,158	3	46
17CS1067	Illumina MiSeq	410.9	241	41	2.21 Mbp	102	38,333	26.93	2,160	3	46
17CS1068-1	Illumina MiSeq	1,215.5	217	110	2.21 Mbp	29	160,528	26.91	2,170	3	46
17CS1068-2	Illumina MiSeq	368.6	201	31	2.21 Mbp	105	32,597	26.94	2,148	3	45
17CS0916-1	Illumina MiSeq	1,019.0	221	94	2.03 Mbp	24	204,435	27.07	2,066	3	46
17CS0916-2	Illumina MiSeq	2,003.3	210	176	2.03 Mbp	25	204,435	27.07	2,066	3	46
16CS0857-1	Illumina MiSeq	812.9	234	80	2.06 Mbp	37	168,312	26.99	2,082	3	46
16CS0861-1	Illumina MiSeq	2,967.4	124	154	2.18 Mbp	64	69,382	26.92	2,149	3	53
17CS1092-1	Illumina MiSeq	860.1	211	76	2.15 Mbp	44	111,096	27.07	2,177	3	45
17CS1092-2	Illumina MiSeq	1,008.8	204	86	2.15 Mbp	46	102,709	27.07	2,174	3	46
17CS1095	Illumina MiSeq	818.2	213	73	2.11 Mbp	48	117,56	27.10	2,127	3	46
16CS0815-2	Illumina MiSeq	841.5	228	80	2.19 Mbp	27	154,166	26.86	2,191	3	46
16CS0820-1	Illumina MiSeq	1,353.3	179	101	2.12 Mbp	21	18,643	26.98	2,117	3	45
16CS0820-2	Illumina MiSeq	1,178.6	200	99	2.12 Mbp	22	199,264	27.05	2,124	3	46
16CS0823-1	Illumina MiSeq	572.6	226	54	2.12 Mbp	23	255,949	27.05	2,122	3	46
16CS0823-2	Illumina MiSeq	563.8	229	54	2.13 Mbp	35	135,934	27.04	2,142	3	46
17CS0965-1	Illumina MiSeq	849.5	218	77	2.43 Mbp	60	95,695	26.84	2,493	3	46
17CS0965-B	Illumina MiSeq	327.8	227	31	2.18 Mbp	147	26,561	26.99	2,166	3	46

### Taxonomic Classification of *A. butzleri* From Germany

**Figure 1:**
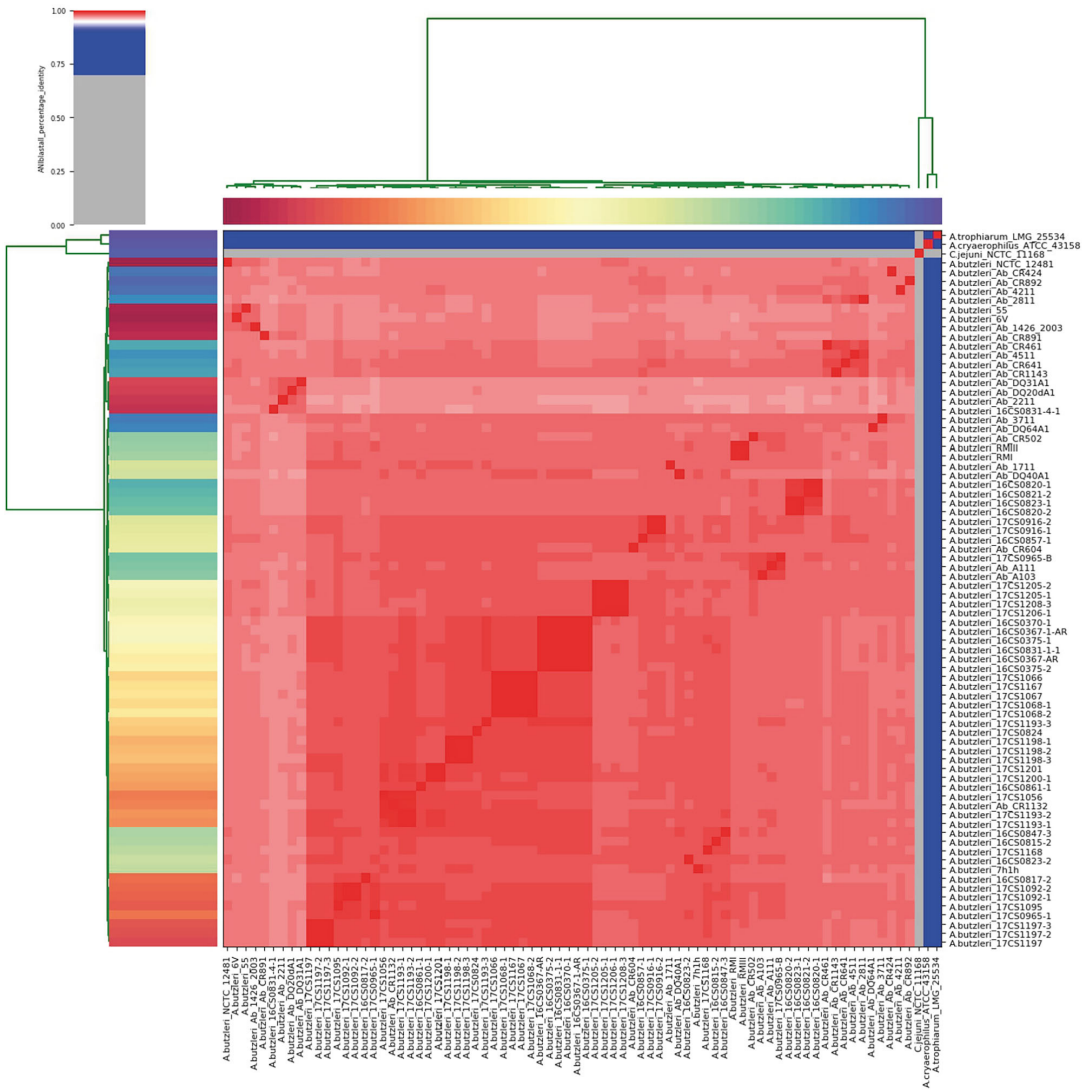
Results of the ANI analysis. The cells in the heatmap corresponding to an ANI value of 95% and above are colored in red, indicating that the associated strains belong to the same species. Strains that do not belong to the same species are colored in blue. The dendrograms (above and on the left side), representing hierarchical clustering of the analysis results in two dimensions, were constructed by the simple linkage of the ANI percentage identities, and correspond to the results of the clustering of the ANI values between the used strains.

The German *A. butzleri* strains sequenced herein were taxonomically classified at species level, based on their reads, with an average of 89.9% (range: 72.2–94.7%) as *A. butzleri* using the bioinformatic tool Kraken2. To confirm this result, ANI was calculated between each genome pair based on the whole-genome sequences. The analysis showed that the German *A. butzleri* strains were highly similar (>95%) to the other European *A. butzleri* strains used in this study, confirming that those genomes belong to the same species (Goris et al., 2007; Richter and Rossello-Mora, 2009) (Figure 1, Supplementary Table 2). All *A. butzleri* strains (*n* = 74) had an average pairwise ANI value of 97.9% (range: 96.75 to 100.0%) and shared a mean nucleotide identity of 97.5% (range: 97.15 to 97.82%) with the *A. butzleri* reference genome NCTC 12481^T^. The closest relative for all 74 *A. butzleri* genomes was *A. cryaerophilus* ATCC 43158^T^ with a mean ANI value of 78.09%. Interestingly, all *A. butzleri* strains were also closely related to *A. trophiarum* LMG 25534^T^ (ANI of 77.22%). The analysis also showed that *A. butzleri* genomes had less than 67% nucleotide identity with *C. jejuni* subsp. *jejuni* NCTC 11168. Additionally, an *in silico* DDH analysis was performed, revealing DDH values above 76% when comparing all 74 *A. butzleri* strains with the reference genome NCTC 12481^T^ (Supplementary Table 3). The recommended standard for delineating distinct species is a DDH threshold of 70% (Wayne et al., 1987; On et al., 2017). *C. jejuni* subsp. *jejuni* NCTC 11168, *A. cryaerophilus* ATCC 43158^T^, and *A. trophiarum* LMG 25534^T^ do not belong to the species *A. butzleri* due to their low DDH values (21.80, 21.70, and 20.90%, respectively). The DDH results supported the results of the ANI analysis because the recommended DDH cut-off value corresponds very well to an ANI value of 95% (Goris et al., 2007; Richter and Rossello-Mora, 2009). Therefore, we concluded that the species of the newly sequenced German strains had been correctly identified as *A. butzleri*.

### Phylogenetic Relatedness of the German *A. butzleri* Strains

**Figure 2:**
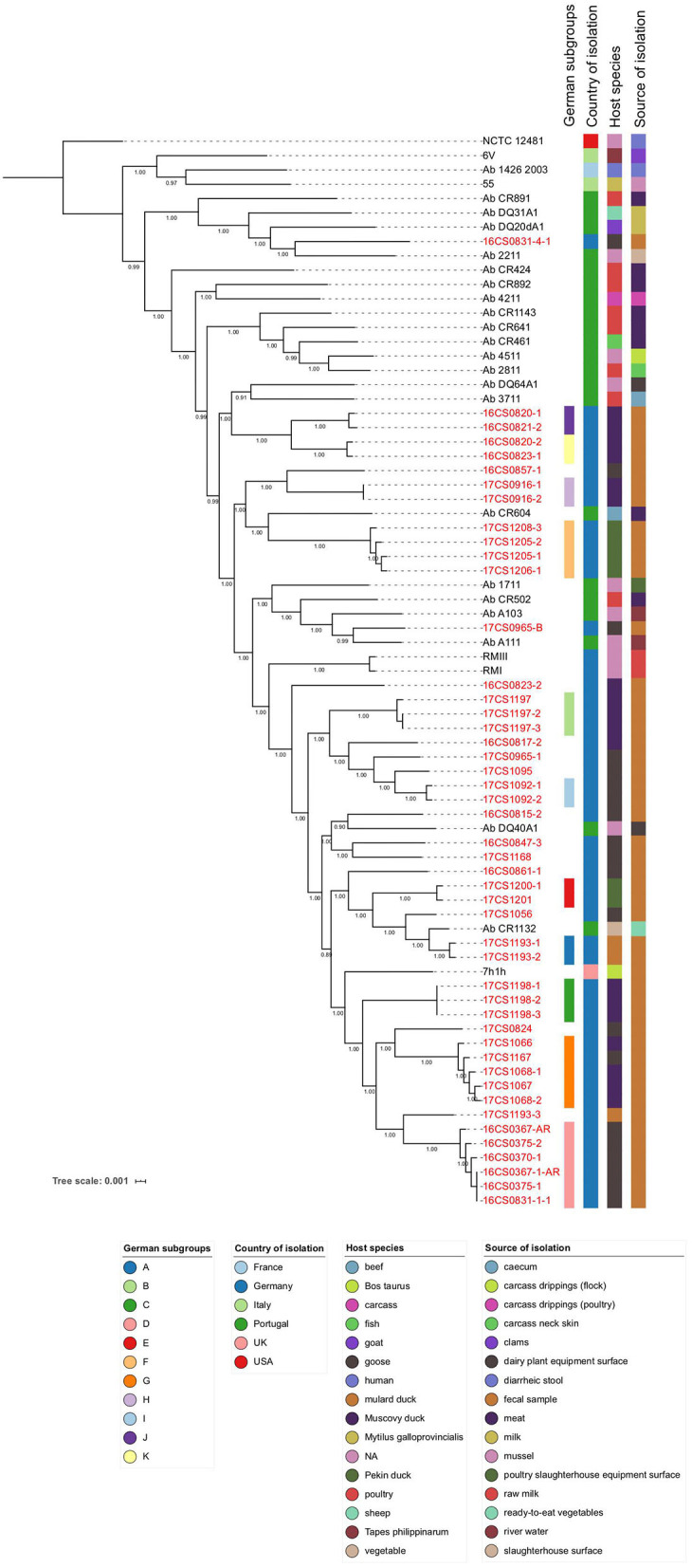
Core-genome based phylogenetic tree involving 75 *A. butzleri* strains with associated metadata. All 47 German *A. butzleri* strains from our collection are highlighted in red. Of those, 33 strains formed 11 subgroups (A–K). Bootstrap values are depicted on the branches.

Figure 2 visualizes the phylogenetic tree based on the core genome alignment (including all *A. butzleri* genomes) done by Roary, which has been used before for this species (Pérez-Cataluña et al., 2018b). Most of the tree branches present high bootstrap values (average: 0.97; range: 0.89–1.00). The identified core genome consists of 1,295 genes corresponding to a size of ~1.18 Mbp, which represents half of the average *A. butzleri* genome. We observed that after adding about 50 genomes, the size of the core genome did not decrease any further (data not shown). The *A. butzleri* accessory genome comprised 10,435 genes. These findings lead to the conclusion that *A. butzleri* harbors an open pan-genome with a total of 11,730 genes. This result is comparable to a previous study (Isidro et al., 2020).

The core genome SNP analysis revealed that the mean pairwise genetic distance between our German *A. butzleri* isolates (*n* = 47) was 10,923 SNPs (range: 0–19,930 SNPs) (Supplementary Table 4). While the majority of our whole-genome sequences has sufficient coverage (>30-fold), four strains (16CS0367-1-AR, 17CS1056, 17CS1205-1, 17CS1208-3) have low coverage (23 to 29-fold). This might be an explanation for the differences between the observed SNP-inferred phylogeny and the epidemiology. Recombination might lead to false-positive SNPs but according to Isidro et al. (2020), they do not affect the phylogeny of *A*. butzleri. However, the distances between the genomes are reduced by 30% (Isidro et al., 2020). Thirty-three German strains could be grouped into 11 subgroups (A to K) (Figure 2). In those groups, the individual strains, which had been isolated from a single sample or multiple samples collected in the same water poultry farm, were less than 50 SNPs apart. The remaining German strains (*n* = 14) could not be assigned to any subgroup. Subgroup G included isolates (*n* = 5) that were recovered from fecal samples collected from two different farms in 2017, four from farm D (four samples from ducks), and one from farm C (one sample from a goose). Those isolates were distant to each other by less than 12 SNPs (Supplementary Table 4). The finding of highly similar strains in two different farms may indicate a possible epidemiological relatedness between them. We speculate that this could be due to a common source of animals or personnel. A fifth sample from farm D was collected from a goose in 2017. But the isolate 17CS1056 retrieved from this sample did not group with the other isolates from farm D, which could be due to the low coverage of the whole-genome sequence of this isolate or due to genetic diversity. In five subgroups (A, B, C, H, and I) the isolates were retrieved from the same sample and were less than or equal to 2 SNPs distant from each other, indicating a clonal-relatedness. Although the remaining 5 subgroups (D, E, F, J, and K) included isolates of two or more samples from the same farm, they were distant to each other by less than 35 SNPs. A possible explanation for this result could be that there is a clone circulating in the water poultry farm's herd. Subgroup A contains two isolates (17CS1193-1 and 17CS1193-2) retrieved from a fecal sample of a duck that was collected from farm A in 2017. The third isolate of this sample, 17CS1193-3, is distant by more than 8,500 SNPs to the two strains of subgroup A. This finding might be due to the presence of two *A. butzleri* strains which are not clonally related. High divergence was also observed in two isolates (16CS0831-1-1, 16CS0831-4-1) from farm B, isolated in 2016 from a goose; a distance of 19,445 SNPs was detected. The same applied for four isolates retrieved out of 2 different samples from ducks in 2016 from farm G. While the isolates 16CS0820-1 and 16CS0820-2 were distant by 6,973 SNPs, the other two isolates (16CS0823-1, 16CS0823-2) were distant by 13,490 SNPs. The same farm had been sampled once more in 2017, and from this additional sample (from a goose), two isolates were recovered. These were ~12,580 SNPs distant from each other. This phenomenon has also been described before for *A. cryaerophilus* isolates from German water poultry (Müller et al., 2020b).

Furthermore, the MLST analysis showed that out of 45 German *A. butzleri* strains sequenced herein, only four were assigned to a known sequence type (ST) (Table 3). The remaining 43 strains most likely belong to new STs. Nevertheless, most of the German *A. butzleri* strains assigned to a subgroup have the same MLST profile. Therefore, we hypothesize that strains with the same allelic profile also belong to the same ST. Since January 2019, the PubMLST database for *Aliarcobacter* is no longer curated, therefore, it was not possible to upload the sequences and assign them to new STs. The PubMLST database contains MLST data of 736 *A. butzleri* isolates from 18 countries. Of these, 725 *A. butzleri* strains were assigned to 680 different STs, showing a high genetic diversity of this species. This high genetic diversity of *A. butzleri* may be the reason why we could not assign 43 of the German genomes to a specific ST. In this study, the MLST results of some European strains varied from the MLST results of a previous study which is most likely due to different assembly approaches (Table 3). In fact, when the available assembly data of the European strains were used for the MLST analysis, the same alleles were identified as in the study conducted by Isidro et al. (2020). However, in some genomes (*n* = 10) the accurate assignment was not possible due to the presence of paralogs for the *gly*A and *glt*A loci (i.e., multiple copies in the same genome). Similar observations were made for strains of the species *A. butzleri* and *A. cryaerophilus* (Isidro et al., 2020; Müller et al., 2020b), although the paralogs found here were not identical except for the *glt*A locus. While developing the first MLST scheme for *Aliarcobacter*, Miller et al. (2009) reported the presence of two *gly*A loci, *gly*A1 and *gly*A2. At the end, the *gly*A1 locus was integrated into the scheme due to its discriminatory power. Consequently, the here identified paralogs of the *gly*A locus could represent the alleles of both *gly*A loci. This is supported by the fact that the detected alleles (1; 142) for the *gly*A locus of the *A. butzleri* reference strain NCTC 12481^T^ are identical with the alleles identified by Miller et al. (2009). In that study, allele 1 was assigned to the *gly*A1 locus and allele 142 to the *gly*A2 locus (Miller et al., 2009). We conclude that the *gly*A locus is either not suitable for MLST analysis since it can lead to incorrect allele calling, or new bioinformatic approaches need to be developed to tackle this problem (Isidro et al., 2020; Müller et al., 2020b).

**Table 3:** Results of the MLST analysis of 75 *A. butzleri* strains.

**Strain**	**Subgroup**	***asp*A**	***atp*A**	***gln*A**	***glt*A**	***gly*A**	***pgm***	***tkt***	**ST**
16CS0367-1-AR	D	4	4	40	19	471, 534	194	158	New
16CS0367-AR	D	4	4	40	19	471, 534	194	158	New
16CS0370-1	D	4	4	40	19	471, 534	194	158	New
16CS0375-1	D	4	4	40	19	471, 534	194	158	New
16CS0375-2	D	4	4	40	19	471, 534	194	158	New
16CS0815-2	–	182	4	40	136	413?	211	87	New
16CS0817-2	–	177	39	40	123	~511	194	24	New
16CS0820-1	J	47	217	4	129	~694	123	37	New
16CS0820-2	K	47	217	9	129	~615	123	37	New
16CS0821-2	J	47	217	4	129	694?	123	37	New
16CS0823-1	K	47	217	9	129	~632	123	37	New
16CS0823-2	–	~229	4	34	4	488	102	63	New
16CS0831-1-1	D	4	4	40	19	471, 534	194	158	New
16CS0831-4-1	–	81	69	26	66	182	124	86	205
16CS0847-3	–	4	12	1	19	346	~103	24	New
16CS0857-1	–	20	7	20	7	186	8	7	New
16CS0861-1	–	4	12	1	19	~418	213	160	New
17CS0824	–	4	58	44	19	150	4	89	New
17CS0916-1	H	20	7	20	23	186?	45	37	New
17CS0916-2	H	20	7	20	23	186?	45	37	New
17CS0965-1	–	153	39	1	19	184	11	~9	New
17CS0965-B	–	20	39	11	11	513	26	55	New
17CS1056	–	37	133	44	12	~379	278	160	New
17CS1066	G	4	4	4	4	~139	4	89	New
17CS1067	G	4	4	4	4	3	4	89	17
17CS1068-1	G	4	4	4	4	139	4	89	18
17CS1068-2	G	4	4	4	4	~139	4	89	New
17CS1092-1	I	153	4	40	19	497?	98	158	New
17CS1092-2	I	153	4	40	19	489?	98	158	New
17CS1095	–	153	4	40	19	528?	98	158	New
17CS1167	G	4	4	4	4	139	4	89	18
17CS1168	–	~69	12	9	19	410	290	165	New
17CS1193-1	A	4	4	44	12	376	4	160	New
17CS1193-2	A	4	4	44	12	376	4	160	New
17CS1193-3	–	4	4	51	126	471	20	89	New
17CS1197	B	~15	4	40	19	~677	63	9	New
17CS1197-2	B	~15	4	40	19	~677	63	9	New
17CS1197-3	B	~15	4	40	19	~677	63	9	New
17CS1198-1	C	4	4	4	19	351	219	158	New
17CS1198-2	C	4	4	4	19	351	219	158	New
17CS1198-3	C	4	4	4	19	351	219	158	New
17CS1200-1	E	41	133	51	19	510	4	160	New
17CS1201	E	41	133	51	19	510	4	160	New
17CS1205-1	F	37	23	7	~165	659	26	New	New
17CS1205-2	F	37	23	7	~165	659	26	New	New
17CS1206-1	F	37	23	7	~165	659	26	New	New
17CS1208-3	F	37	23	7	~165	659	26	New	New
Ab_CR1143	–	23	5	24	44	112	35	55	New
Ab_3711	–	20	2	1	~15	~449	71	165	New
Ab_CR1132	–	240	133	40	12	541	278	160	519
Ab_1711	–	13	4	40	15,15	**~536** (545)	102	158	New
Ab_CR461	–	5	5	24	27	80	62	20	New
Ab_4511	–	23	44	24	44	112	35	20	510
Ab_2211	–	234	15	26	164	375	260	176	460
Ab_2811	–	23	44	24	2	**769?** (137)	62	20	**New** (107)
Ab_A103	–	20	~39	11	11	98	87	178	New
Ab_CR424	–	23	7	33	38	**156** (~745)	~120	174	New
Ab_CR641	–	23	44	24	15	124	55	20	108
Ab_DQ20dA1	–	59	47	26	53	~205	88	60	New
Ab_DQ64A1	–	10	20	11	19	192	**123** (134)	11	New
Ab_CR604	–	26	22	4	44	~24	55	165	New
Ab_4211	–	20	25	7	2	**~595** (~465)	32	2	New
Ab_DQ40A1	–	4	164	40	19	**418** (528)	102	4	New
Ab_1426_2003	–	8	8	8	8	**191** (9)	9	8	**New** (47)
Ab_CR502	–	20	39	11	15	**~103** (~666)	~26	2	New
Ab_A111	–	20	12	11	19	**112?** (~692)	127	88	New
Ab_CR892	–	10	14	41	30	**747?** (~602)	~263	~31	New
Ab_CR891	–	21	22	21	24	48	27	25	94
Ab_DQ31A1	–	55	45	26	48	113	85	59	172
7h1h	–	150	4	1	122	~370	194	52	New
6V	–	236	161	1	183	521	296	207	537
55	–	332	8	1	166	685	367	292	675
RMIII	–	5	5	5	15	66,176	11	10	New
RMI	–	5	5	5	15	66,176	11	10	New
NCTC 12481	–	1	1	1	1	1,142	1	1	New

*The allele loci that do not agree with previous results are written in bold. The alleles identified by Isidro et al. (2020) are shown in brackets. (~ = novel full length allele similar to a known allele; ? = allele partial matches to a known allele)*.

In summary, our results indicate a high genetic diversity among *A. butzleri* strains, although the sample pool of German strains was limited to one federal state (Thuringia, Germany), water poultry as host, and a short study period (2 years). Since similar isolates were identified in geese and ducks (e.g., as in subgroup G), strain diversity within *A. butzleri* seems to be independent of the host species. This was also supported by the overall phylogeny, as it was not possible to identify major clusters related to the host species. The phylogenetic analysis did also not support clustering based on either the isolation source or geographical location of the genomes. Similar observations have been reported before for *A. cryaerophilus* (Müller et al., 2020b).

### Antibiotic Susceptibility Testing

The 45 German *A. butzleri* strains were resistant to cefotaxime (Supplementary Table 1). Resistance to cefotaxime in *A. butzleri* is well-known, and therefore, cefotaxime is often added to selective media to inhibit the growth of accompanying bacteria (Shah et al., 2013; Rathlavath et al., 2017; Fanelli et al., 2019; Müller et al., 2020a). None of the strains was resistant to gentamicin but 26 strains showed intermediate resistance. These findings are in line with earlier studies (Abay et al., 2012; Kayman et al., 2012; Shah et al., 2012b; Ferreira et al., 2013, 2019; Van den Abeele et al., 2016; Rathlavath et al., 2017; Fanelli et al., 2019; Isidro et al., 2020). Our data shows that gentamicin can still be used for the treatment of *Aliarcobacter* infections like it has been suggested before (Ramees et al., 2017; Rathlavath et al., 2017), but the increasing resistance has to be kept in mind. In our study, only two isolates (17CS0916-1, 17CS0916-2) were resistant to ciprofloxacin. This result is consistent with previous studies (Miller et al., 2007; Kayman et al., 2012; Ferreira et al., 2017; Fanelli et al., 2019, 2020), although there are studies which have reported higher resistance rates (Collado and Figueras, 2011; Shah et al., 2012b; Ferreira et al., 2013, 2019; Isidro et al., 2020). From a total of 45 *A. butzleri* isolates tested in this study, 32 were resistant to erythromycin, 26 to doxycycline and 20 to tetracycline. These results are in line with previous studies, but resistance to erythromycin has been described controversially (Miller et al., 2007; Abay et al., 2012; Kayman et al., 2012; Shah et al., 2012b; Van den Abeele et al., 2016; Ferreira et al., 2017; Rathlavath et al., 2017; Fanelli et al., 2019, 2020; Isidro et al., 2020). Similar resistance rates for ciprofloxacin, tetracyclines, and erythromycin have been observed for *Campylobacter* spp. in poultry (Nobile et al., 2013; Marotta et al., 2019). Rising resistance in *Campylobacter* spp. has been connected with the inappropriate use or overuse of antibiotics in animal husbandry (Endtz et al., 1991; Marotta et al., 2019). We assume, that this might also be true for tetracyclines and erythromycin and *Aliarcobacter* spp. as they are admitted for treatment of bacterial infections in poultry in Germany. According to existing studies *A. butzleri* isolates are highly resistant to ampicillin (Abay et al., 2012; Kayman et al., 2012; Shah et al., 2012b; Van den Abeele et al., 2016; Rathlavath et al., 2017; Fanelli et al., 2019, 2020). However, in this study more than half of the *A. butzleri* strains (*n* = 28) were susceptible to ampicillin *in vitro*. Nearly all studied strains were resistant to streptomycin (*n* = 39), although in previous studies *A. butzleri* strains were usually sensitive to streptomycin (Abay et al., 2012; Rathlavath et al., 2017; Fanelli et al., 2019, 2020). This indicates an increase in resistance. Therefore, we do not recommend the use of streptomycin for the treatment of *A. butzleri* infections in both veterinary and human medicine.

In previous studies, the antimicrobial susceptibility of *A. butzleri* has been determined with different methods e.g., disk diffusion, broth microdilution, or agar diffusion. However, according to a study conducted by Van den Abeele et al. (2016), the gradient strip method should be preferred over the disk diffusion method. Since both methods' agreement stands at only 60%, our results can be compared with those of earlier studies to a limited extent. This shows the need for a standardized method for antimicrobial susceptibility testing of *Aliarcobacter* spp., and for the interpretation of the results as already noticed by other authors (Ferreira et al., 2016; Ramees et al., 2017; Riesenberg et al., 2017).

### *In silico* Prediction of Antimicrobial Resistance Genes

To determine the underlying genotypic antimicrobial resistance (AMR) profile, we screened all 75 *A. butzleri* genomes with a custom-made database (ARCO_IBIZ_AMR) as described previously (Müller et al., 2020a). This database contains 92 putative AMR genes and 27 putative heavy metal resistance genes.

The survey revealed that all tested genomes contained an average of 80 potential antimicrobial and heavy metal resistance genes. Strain NCTC 12481^T^ harbored most of the genes (*n* = 104) and strain Ab_3711 the fewest (*n* = 66) (Supplementary Table 5).

Out of 19 efflux pumps (EP) that have been described before for *A. butzleri* (Isidro et al., 2020), 17 EP systems were found in the *A. butzleri* genomes tested in this study. The two missing efflux pump systems are EP18 and EP19 [both belong to the resistance-nodulation-division (RND) family]. Eight EPs were present in all genomes: (a) EP2, EP12, EP13, and EP14 [all members of the major facilitator superfamily (MFS)]; (b) EP5, EP6, and EP10 [belonging to the ATP-binding cassette (ABC) family]; and (c) EP8 [belongs to the small multidrug resistance (SMR) family]. This result is concordant with those of previous studies (Isidro et al., 2020; Müller et al., 2020a). The remaining nine EPs belong to the ABC (EP3), RND (EP4, EP7, EP11, EP15, EP16), and MFS families (EP9, EP17) and were present in 16 *A. butzleri* genomes. These findings showed that *A. butzleri* harbors all major families of efflux transporters which are present in prokaryotes except for the multidrug and toxic efflux (MATE) family (Webber and Piddock, 2003). Although they are classified into different families, EPs can confer multidrug resistance as they can export a variety of different substrates (Van Bambeke et al., 2000; Sun et al., 2014; Alcalde-Rico et al., 2016). It is worth mentioning that EP5, EP6, EP8, EP9, EP12, EP13, EP14, and EP17 each consist of only one gene. Hereby, EP9, EP14, and EP17 are of particular interest. Both, EP14 and EP17, contain the same gene, the *bcr* gene which is associated with resistance to sulfonamides and bicyclomycin in *Escherichia coli* (*E. coli*) (Nichols and Guay, 1989). EP9 contains the *fsr* gene that confers resistance to fosmidomycin (Fanelli et al., 2020). The presence of an EP does not necessarily mean that resistance to a particular antibiotic is also present; the resistance rather depends on the expression level of the EP (Alcalde-Rico et al., 2016).

Type 1 secretion systems (T1SS), which are responsible for the transport of unfolded substrates across the inner and outer membrane, are usually found in Gram-negative bacteria (Green and Mecsas, 2016). The putative T1SS described for *A. butzleri* (Isidro et al., 2020), was only present in six strains.

The analysis of potential antibiotic determinants revealed that all tested genomes carried additional putative multidrug export ATP-binding/permease proteins (RM4018p_11130, *ybi*T1, *ylm*A, *mac*B1) and parts of putative EP systems (*acr*B, *tol*C). The outer membrane protein gene, *opr*F3, and the putative multidrug export ATP-binding/permease protein, RM4018p_04700, were also present in all *A. butzleri* genomes except strain 16CS0817-2.

Resistance to tetracyclines is probably due to efflux pump mechanisms, ribosomal protection and enzymatic inactivation (Grossman, 2016). Although the *tet*A gene—encoding the tetracycline EP as described for *Campylobacter* spp. (Shen et al., 2018)—was present in 37 genomes, the phenotype did not correspond to the genotype as both, sensible and resistant isolates carried this gene (Table 4). However, as mentioned above, the phenotype may depend on the expression level of the EP. Ribosomal protection proteins encoded by, for example the *tet*(O) gene, were not present, which is not surprising as they are usually located on plasmids (Elhadidy et al., 2020). Although the plasmid prediction in this study revealed the possible presence of a known *A. butzleri* plasmid in seven strains (Supplementary Table 6), none of the suggested plasmid carried a potential resistance or virulence-associated gene. Therefore, *A. butzleri* strains either carry a hitherto unknown plasmid or harbor an unknown resistance mechanism against doxycycline and tetracycline. Further studies on this topic are needed in the near future.

**Table 4:** Genotype-phenotype correlations regarding antibiotic resistance detected in this study.

**Strain**	**DC**	***tetA_gene***	**TET**	**CIP**	gyrA_gene^*^	**AMP**	***bla2_gene***	***bla3_gene***	***hcpC_gene***	***mrdA_gene***	***pbpB_gene***	***pbpF_gene***	**CTX**	**Urease assay**	**Urease cluster**
16CS0847-3	S		S	S		S							R	+	
17CS0824	R		R	S		S							R	+	
17CS1193-1	S		S	S		R							R	+	
17CS1193-2	S		S	S		R							R	+	
17CS1193-3	S		S	S		S							R	+	
17CS1197	R		R	S		S							R	+	
17CS1197-2	R		R	S		S							R	+	
17CS1197-3	R		R	S		S							R	+	
17CS1198-1	S		S	S		S							R	+	
17CS1198-2	S		S	S		S							R	+	
17CS1198-3	S		S	S		S							R	Ø	
16CS0367-AR	R		R	S		S							R	+	
16CS0367-1-AR	R		R	S		S							R	+	
16CS0370-1	R		R	S		S							R	+	
16CS0375-1	R		R	S		S							R	+	
16CS0375-2	R		R	S		S							R	+	
16CS0831-1-1	R		R	S		R							R	+	
16CS0831-4-1	R		R	S		R							R	Ø	
17CS1200-1	S		S	S		S							R	+	
17CS1201	S		S	S		S							R	+	
17CS1205-1	S		S	S		R							R	+	
17CS1205-2	R		S	S		R							R	+	
17CS1206-1	R		S	S		R							R	+	
17CS1208-3	R		S	S		R							R	+	
17CS1167	R		S	S		S							R	+	
17CS1168	S		S	S		S							R	+	
17CS1056	S		S	S		S							R	+	
17CS1066	S		S	S		R							R	+	
17CS1067	S		S	S		R							R	+	
17CS1068-1	R		R	S		R							R	+	
17CS1068-2	R		R	S		R							R	+	
17CS0916-1	S		R	R		R							R	+	
17CS0916-2	R		R	R		R							R	+	
16CS0857-1	R		R	S		R							R	+	
16CS0861-1	R		S	S		S							R	+	
17CS1092-1	R		R	S		S							R	+	
17CS1092-2	S		S	S		S							R	+	
17CS1095	S		S	S		S							R	+	
16CS0815-2	S		S	S		S							R	+	
16CS0817-2	S		S	S		S							R	+	
16CS0820-1	R		R	S		R							R	+	
16CS0820-2	R		R	S		S							R	+	
16CS0821-2	R		R	S		S							R	+	
16CS0823-1	R		R	S		R							R	+	
16CS0823-2	R		S	S		S							R	+	
17CS0965-1	S		S	S		S							R	+	
17CS0965-B	R		S	S		S							R	+	

*The determined phenotype of the 47 German strains is presented next to the associated genotype, their presence/absence is marked by colored/ white boxes*.

* *Presence of the mutation Thr-85-Ile*.

*R, resistant phenotype; S, susceptible phenotype; +, positive urease assay; Ø, negative urease assay; DC, doxycycline; TET, tetracycline; CIP, ciprofloxacin; AMP, ampicillin; CTX, cefotaxime*.

In this study, five genes which might cause phenotypic resistance to cefotaxime were identified in all genomes: *bla*2 (putative metallo-hydrolase), *hcp*C (putative beta-lactamase), *mrd*A, *pbp*B, and *pbp*F (all penicillin-binding proteins). Penicillin-binding proteins are the target of beta-lactam antibiotics and are therefore not a resistance mechanism as such, unless they have a low affinity for beta-lactams (Macheboeuf et al., 2006; Zapun et al., 2008). As already described by Georgopapadakou (1993), resistance to beta-lactams in Gram-negative bacteria is the result of a combination of the presence and activity of beta-lactamase genes and penicillin-binding proteins as well as reduced membrane permeability. However, this would contradict the phenotype determined against ampicillin (Table 4). Isidro et al. (2020) noticed a strong correlation between the ampicillin resistance and the presence of the *bla*3 gene (OXA-15 beta-lactamase). This hypothesis is partly supported by the results of this study. Here, only two strains (16CS0831-1-1, 16CS0820-1) that did not carry the *bla*3 gene were resistant to ampicillin, and of 33 genomes carrying the *bla*3 gene, only four isolates (17CS1167, 16CS0820-2, Ab_CR1132, Ab_DQ64A1) were susceptible to ampicillin.

As reported previously, resistance to ciprofloxacin in *Aliarcobacter* spp. is due to a point mutation (C254T) in the quinolone resistance-determining region (QRDR) at position 254 of the *gyr*A gene (Abdelbaqi et al., 2007). This transition leads to the substitution of threonine to isoleucine (Thr-85-Ile). In this study, 11 *A. butzleri* genomes showed this mutation, two German strains (17CS0916-1 and 17CS0916-2) and nine Portuguese strains (Ab_1711, Ab_2811, Ab_3711, Ab_4211, Ab_A103, Ab_CR1143, Ab_CR502, Ab_CR892, and Ab_DQ20dA1). Those *A. butzleri* strains were also phenotypically resistant to ciprofloxacin (Table 4, Supplementary Table 1). This result is concordant with earlier studies, although higher resistance rates have been reported (Fera et al., 2003; Ferreira et al., 2013, 2017; Van den Abeele et al., 2016; Rathlavath et al., 2017; Vicente-Martins et al., 2018).

Point mutations in the 23S rRNA gene and amino acid changes in the *rpl*D and/or *rpl*V gene are considered to be the cause of resistance to erythromycin, as described previously for *Campylobacter* spp. (Alfredson and Korolik, 2007; Perez-Boto et al., 2010; Bolinger and Kathariou, 2017; Shen et al., 2018; Elhadidy et al., 2020). All used genomes were screened for modifications, but none was detected in any strain. Therefore, the resistance mechanism in *A. butzleri* needs to be different. In a previous study, it has been hypothesized that the protein size of the regulator TetR (RM4018p_22360), part of EP16, might correlate with the resistance to erythromycin. Truncating mutations in the *tet*R gene could lead to overexpression of EP16 and thus to increased excretion of erythromycin (Isidro et al., 2020). In another study, the involvement of EP3 has been discussed because it contains two macrolide export proteins (encoded by *mac*A1 and *mac*B2 gene) (Fanelli et al., 2019). Although the presence of those export proteins could explain the phenotype of the resistant isolates, it contradicts the phenotype of the susceptible isolates. The fact that these genes were present but not expressed, might explain the susceptible phenotype.

Furthermore, the screening for other antibiotic determinants identified additional genes in all isolates that are potentially responsible for resistance to certain antibiotics: *arn*B and *ept*A (conferring resistance to polymyxin), and *rlm*N (resistance to various classes of antibiotics) (Fanelli et al., 2019, 2020). The two genes, *cat*3 and *wbp*D, which confer resistance to chloramphenicol were detected in 11 and 27 genomes, respectively. While nine isolates carried three *hip*A genes (*hip*A2, *hip*A3, and *hip*A4), was the *hip*A1 gene only present in two and the *hip*A2 gene in three genomes. The *hip*A genes encode a serine/threonine-protein kinase—a toxic component of the type II toxin-antitoxin system—and are suspected to be involved in multidrug resistance (Schumacher et al., 2009).

### *In silico* Prediction of Heavy Metal Resistance

The survey for heavy metal resistance genes revealed the presence of a putative cluster of genes coding for arsenic resistance in all isolates (Supplementary Table 5). This arsenic cluster consists of four genes: *ars*B (arsenic pump membrane protein), *ars*C1 (arsenate reductase), *ars*C2 (glutaredoxin arsenate reductase), and an arsenic resistance protein (ABU_RS02800). Simple arsenic clusters or *ars* operons have been reported for various Gram-negative bacteria (Ben Fekih et al., 2018). Also a putative copper cluster was found in 59 genomes, consisting of six genes: *cop*A1 (copper-exporting P-type ATPase A), *cop*A2 (putative copper-importing P-type ATPase A), *cop*R (transcription activator protein), *cop*Z (copper chaperone), *cso*R (copper-sensing transcriptional repressor), and *cus*S (sensor kinase). Furthermore, a molybdate cluster (ABC-type transport system) that has been described for *E. coli* and *Staphylococcus carnosus*, was present in all strains (Rech et al., 1995; Neubauer et al., 1999). However, the cluster was incomplete because the *mod*C gene (encoding the cytoplasmic ATPase) was not present. Instead of the *mod*C gene, we identified the *mop*A gene (regulator of *mod*ABC) which has been described in *Rhodobacter capsulatus* (Wiethaus et al., 2006). These findings are mostly in line with the results of previous studies (Fanelli et al., 2019, 2020).

The export mechanism for cadmium, zinc, and cobalt represented by *cad*A (a cadmium, zinc and cobalt transporting ATPase) and *czc*D (a cadmium, cobalt and zinc/H^+^-K^+^ antiporter) was present in all genomes except for Ab_3711. This is concordant with previous studies (Fanelli et al., 2019, 2020). The genes *czc*A and *czc*B, encoding cobalt-zinc-cadmium resistance proteins, were found in eight and 10 strains, respectively. Interestingly, three genes (*czc*R1, *czc*R2 and *czc*R3) responsible for the transcription activator protein CzcR were found in all strains, while the *czc*R4 gene was only present in 22 genomes.

Furthermore, all *A. butzleri* genomes carried four additional transporters: a mercuric transporter (*mer*T), a zinc transporter (*znt*B), a putative manganese/zinc-exporting P-type ATPase (*ctp*C) and a magnesium and cobalt efflux protein (*cor*C) (Fanelli et al., 2020).

The detection of heavy metal resistance clusters/transporters was expected, as the co-resistance and co-selection of AMR and heavy metal resistance, as well as the risk of simultaneous horizontal transmission between bacteria, is driven by their genetic linkage (Bengtsson-Palme and Larsson, 2015; Fanelli et al., 2019, 2020; Zhao et al., 2019). However, this also means that in an environment polluted with heavy metals, this mechanism will lead to the promotion of antibiotic resistance even without the presence of antimicrobials (Fanelli et al., 2020).

### *In silico* Virulence Profiling

The virulence-associated genes of all 75 *A. butzleri* genomes were determined using the database ARCO_IBIZ_VIRULENCE (Müller et al., 2020a). This database can identify 148 virulence-associated genes, including flagellar genes (*n* = 36), chemotaxis system genes (*n* = 8), urease cluster genes (*n* = 6), putative capsule cluster genes (*n* = 7), type IV secretion system (T4SS) genes (*n* = 55), lipid A cluster genes (*n* = 12), and other virulence determinants (*n* = 24) described in earlier studies (Fanelli et al., 2019, 2020; Isidro et al., 2020; Müller et al., 2020a). The analysis showed that all investigated genomes carried an average of 78 putative virulence-associated genes (Supplementary Table 7). While strain 17CS0965-1 had most of the genes (*n* = 87), strains Ab_2811 and Ab_A103 had the fewest (*n* = 71).

The detection of virulence-associated genes was first reported in the context of the description of the complete genome sequence of *A. butzleri* RM4018 (Miller et al., 2007). In that study, Miller and his colleagues discovered homologs of the virulence genes *cad*F, *cia*B, *cj*1349, *mvi*N, *pld*A, and *tly*A of *C*. *jejuni* as well as four other virulence determinants (*irg*A, *iro*E, *hec*A, *hec*B) in *A. butzleri* RM4018 (Miller et al., 2007). Of these virulence-associated genes, the following genes associated with cell adhesion and invasion were present in all 75 genomes: *cia*B (host cell invasion protein), *opr*F2 (fibronectin-binding protein), *cj*1349 (fibronectin-binding protein), *mur*J (integral membrane protein of murein biosynthesis), *pld*A (outer membrane phospholipase A), *tly*A (hemolysin), and additionally *deg*P (chaperone involved in adhesion folding; Isidro et al., 2020). These results are largely consistent with those of previous studies (Douidah et al., 2012; Karadas et al., 2013; Rathlavath et al., 2017; Fanelli et al., 2019, 2020; Parisi et al., 2019; Isidro et al., 2020). The virulence determinants *cir*A1 and *bes*A, which have been associated with the uropathogenicity in *E. coli*, were detected in 11 and 57 genomes, respectively. This is also consistent with earlier studies, as both genes are found less frequently in *A. butzleri* strains (Miller et al., 2007; Fanelli et al., 2019, 2020; Isidro et al., 2020). Three additional *cir*A genes (*cir*A2, *cir*A3, *cir*A4) were identified in some genomes. While *cir*A2 and *cir*A4 had the same annotation made by Prokka as *cir*A1—colicin I receptor—suggesting that both may be involved in iron uptake (Griggs et al., 1987), the *cir*A3 gene represents the former *cfr*B gene, which is involved in iron absorption (Isidro et al., 2020). The *fur* gene, which regulates the ferric uptake, was found in all tested genomes (Griggs et al., 1987; Isidro et al., 2020). According to existing studies, the genes *cdi*A (filamentous hemagglutinin) and *shl*B (hemolysin transporter protein) are rarely found. However, there are no consistent data on the presence of *shl*B (Douidah et al., 2012; Karadas et al., 2013; Rathlavath et al., 2017; Fanelli et al., 2019, 2020; Isidro et al., 2020). This is supported by the results of our study since *cdi*A was found in only two genomes (NCTC 12481^T^, Ab_DQ20dA1) and *shl*B in 11 genomes, respectively.

*A. butzleri* is a motile bacterium due to the polar flagellum. Therefore, the presence of flagellar genes was expected. All 36 flagellar genes were present in 62 genomes. Among the remaining 13 genomes, the *fla*A and *fla*B genes (both coding for flagellin) were absent in seven genomes, while in six genomes, only the *fla*A gene was missing. Similar results have been reported in previous studies (Fanelli et al., 2019; Isidro et al., 2020). The absence of both *fla* genes could have consequences for the assembly and/or function of the flagellum (Isidro et al., 2020).

Each of the 75 tested genomes contained a complete chemotaxis system. This is consistent with the results of earlier studies (Miller et al., 2007; Isidro et al., 2020). Of the two chemotaxis-associated genes, the *lux*S gene was present in all genomes, while the *ccp* gene was detected in only four genomes (Isidro et al., 2020).

The complete urease cluster was detected in 59 *A. butzleri* genomes. Forty-seven German *A. butzleri* isolates (45 strains from this study and two strains from a previous study of the authors; Müller et al., 2020a) underwent a functional urease test. The assay revealed that 45 strains were urease-positive. The urease-negative phenotype of strain 16CS0831-4-1 was consistent with the genotype (Table 4) because of the absence of the urease cluster in this strain. While the urease cluster was present in strain 17CS1198-3, the urease assay of this strain was negative. A detailed examination of the urease cluster of this isolate showed several changes in the amino acid sequence of the *ure*E gene (encoding the urease accessory protein) which could justify the observed discrepancy. Although 17CS0965-B did not carry the urease cluster, this strain was phenotypically positive. So far, we do not have a sufficient explanation for this finding. Overall, these results are concordant with previous results (Isidro et al., 2020). Consequently, we hypothesize that there is a correlation between the occurrence of the urease cluster and the urease-positive phenotype. While the ability of *A. butzleri* to metabolize urea is well-known (Miller et al., 2007), the presence of the urease enzyme may also be a threat to a potential host. Due to the degradation of urea to ammonium and carbon dioxide, *A. butzleri* might be able to survive in an acidic environment as observed for *H. pylori* (Gupta et al., 2019).

As part of lipopolysaccharide (LPS), an endotoxin in Gram-negative bacteria, lipid A is recognized by the innate immune system and can trigger a strong immune response in humans and animals (Emiola et al., 2014). Therefore, it should be considered as an important virulence factor. We highlight the detection of a complete putative lipid A cluster in all 75 *A. butzleri* genomes. The putative lipid A cluster contains eight genes that are necessary for the biosynthesis of lipid A: *lpx*A, *lpx*B, *lpx*C, *lpx*D, *lpx*H, *lpx*K, *lpx*P, and *waa*A (Opiyo et al., 2010; Emiola et al., 2014; Steimle et al., 2016). Although a ninth gene, the *lpx*M gene (encoding myristoyltransferase), is described, its presence is usually not essential (Raetz et al., 2009; Emiola et al., 2014; Steimle et al., 2016). The presence of the *lpx*P gene (a paralogue of *lpx*L) in all genomes could explain the adaptation of growth to low temperatures and the survival of *A. butzleri* outside a host because the palmitoleolytransferase encoded by the *lpx*P gene is induced by low temperatures (Carty et al., 1999; Opiyo et al., 2010). Previous studies reported that some Gram-negative bacteria have the ability to change their lipid A structure (Ernst et al., 2001; Ramachandran, 2014; Steimle et al., 2016). These changes are regulated by a two-component system called PhoP-PhoQ and can lead, for example, to increased resistance to cationic antimicrobial peptides and reduced receptor recognition (Ramachandran, 2014). Two genomes, NCTC 12481^T^ and 6V, carried the two-component system consisting of three *pho*P genes (*pho*P1, *pho*P2, and *pho*P3) and the *pho*Q gene. While the PhoP-PhoQ system was detected in four strains (16CS0831-4-1, Ab_2211, Ab_DQ20dA1, and Ab_DQ31A1) without the *pho*P2 gene, *pho*P3, and *pho*Q were the only genes found in 17CS0965-1. Since no data were available in the current literature on how many *pho*P genes are necessary for the functionality of the PhoP-PhoQ system, it remains unclear if these seven strains might be able to modify their lipid A structure. All the other genomes had at least one *pho*P gene (*pho*P3) but no *pho*Q gene, suggesting that the two-component system was not functional. These *A. butzleri* strains were therefore either unable to modify their lipid A structure or used a hitherto unknown mechanism.

In this study, the genes coding for heptosyltransferases I and II, namely *rfa*C and *rfa*F, were identified in all 75 *A. butzleri* genomes. These heptosyltransferases are possibly responsible for the assembly and the phosphorylation of the inner-core region of the LPS (Rovetto et al., 2017; Fanelli et al., 2020). Although heptosyltransferases are not essential, their absence may affect the structure of the outer membrane of Gram-negative bacteria, and thus lead to a rough phenotype (Gronow et al., 2000).

Furthermore, the following genes were detected in all analyzed genomes: (a) *cvf* B (conserved virulence factor B), which is known to regulate the expression of virulence factors in *Staphylococcus aureus* (Matsumoto et al., 2007); (b) *voc* (virulence protein); and (c) *vir*F (virulence regulator transcription activator), which has been associated with the regulation of plasmid-transmitted virulence genes in *Shigella flexneri* (Adler et al., 1989; Tobe et al., 1993). In contrast, the *bvg*S gene (virulence sensor protein) was only present in six *A. butzleri* strains.

## Conclusion

In this study, 45 *A. butzleri* strains isolated from seven water poultry farms in Thuringia, Germany, were sequenced and correctly identified as *Aliarcobacter butzleri*.

We observed a high genetic diversity between the German *A. butzleri* strains, even though the isolates were limited to one federal state (Thuringia), a certain host (water poultry), and a short study period (2 years). However, the phylogenetic analysis did not support clustering based on the isolation source or geographical location of the strains.

Here, we were able to identify a comprehensive profile of potential resistance and virulence-associated genes in all investigated *A. butzleri* genomes. The antimicrobial genotype, however, only corresponds partially to the determined phenotype. Therefore, phenotypic antimicrobial susceptibility testing remains necessary and important. A strong correlation was found between the presence of the *gyr*A mutation (Thr-85-Ile) and the phenotypic ciprofloxacin resistance, as well as between the presence of the urease cluster and urease-positive phenotype. Only a partial correlation was observed between the presence of the *bla*3 gene and ampicillin resistance. In addition, we would like to highlight the identification of a lipid A cluster in all studied genomes, which may allow some strains a certain amount of flexibility to the host's immune response due to the ability to modify their lipid A structure.

## Data Availability Statement

The original contributions generated for this study are publicly available. This data can be found here: DDBJ/ENA/GenBank; BioProject: PRJNA665330, and PRJNA575341. Publicly available datasets were analyzed in this study. This data can be found here: DDBJ/ENA/GenBank; BioProject: PRJEB6403, PRJNA489574, PRJNA489609, PRJNA637480, PRJNA67167, and PRJEB34441.

## Ethics Statement

Ethical review and approval was not required for the animal study because there were no experiments with animals as defined by the German Animal Protection Law (Tierschutzgesetz) and the Animal Welfare Laboratory Animal Regulation (Tierschutz-Versuchstierverordnung). Written informed consent was obtained from the owners for the participation of their animals in this study.

## Author Contributions

EM and HT conceived the work. IH provided the strains and metadata. EM and IH performed antimicrobial susceptibility testing. EM performed the urease test and bioinformatic analyses and wrote the manuscript. HH, JL, and HT supervised the data interpretation. All authors contributed to the revision of the manuscript, read and approved the submitted manuscript.

## Conflict of Interest

The authors declare that the research was conducted in the absence of any commercial or financial relationships that could be construed as a potential conflict of interest.
